# Investigation of the usability of conebeam CT data sets for dose calculation

**DOI:** 10.1186/1748-717X-3-42

**Published:** 2008-12-16

**Authors:** Anne Richter, Qiaoqiao Hu, Doreen Steglich, Kurt Baier, Jürgen Wilbert, Matthias Guckenberger, Michael Flentje

**Affiliations:** 1 Julius-Maximilians-University, Department of Radiation Oncology, Wuerzburg, Germany

## Abstract

**Background:**

To investigate the feasibility and accuracy of dose calculation in cone beam CT (CBCT) data sets.

**Methods:**

Kilovoltage CBCT images were acquired with the Elekta XVI system, CT studies generated with a conventional multi-slice CT scanner (Siemens Somatom Sensation Open) served as reference images. Material specific volumes of interest (VOI) were defined for commercial CT Phantoms (CATPhan^® ^and Gammex RMI^®^) and CT values were evaluated in CT and CBCT images. For CBCT imaging, the influence of image acquisition parameters such as tube voltage, with or without filter (F1 or F0) and collimation on the CT values was investigated. CBCT images of 33 patients (pelvis n = 11, thorax n = 11, head n = 11) were compared with corresponding planning CT studies. Dose distributions for three different treatment plans were calculated in CT and CBCT images and differences were evaluated. Four different correction strategies to match CT values (HU) and density (D) in CBCT images were analysed: standard CT HU-D table without adjustment for CBCT; phantom based HU-D tables; patient group based HU-D tables (pelvis, thorax, head); and patient specific HU-D tables.

**Results:**

CT values in the CBCT images of the CATPhan^® ^were highly variable depending on the image acquisition parameters: a mean difference of 564 HU ± 377 HU was calculated between CT values determined from the planning CT and CBCT images. Hence, two protocols were selected for CBCT imaging in the further part of the study and HU-D tables were always specific for these protocols (pelvis and thorax with M20F1 filter, 120 kV; head S10F0 no filter, 100 kV). For dose calculation in real patient CBCT images, the largest differences between CT and CBCT were observed for the standard CT HU-D table: differences were 8.0% ± 5.7%, 10.9% ± 6.8% and 14.5% ± 10.4% respectively for pelvis, thorax and head patients using clinical treatment plans. The use of patient and group based HU-D tables resulted in small dose differences between planning CT and CBCT: 0.9% ± 0.9%, 1.8% ± 1.6%, 1.5% ± 2.5% for pelvis, thorax and head patients, respectively. The application of the phantom based HU-D table was acceptable for the head patients but larger deviations were determined for the pelvis and thorax patient populations.

**Conclusion:**

The generation of three HU-D tables specific for the anatomical regions pelvis, thorax and head and specific for the corresponding CBCT image acquisition parameters resulted in accurate dose calculation in CBCT images. Once these HU-D tables are created, direct dose calculation on CBCT datasets is possible without the need of a reference CT images for pixel value calibration.

## Background

Recently, cone-beam CT (CBCT) technology found on linear accelerators has enabled three dimensional imaging of the patient in the treatment position [1]. These images are most frequently used for image-guidance: positioning of the patient or target position is evaluated by a comparison of the CBCT with the planning CT [2-6]. Set-up errors are then corrected by shifts of the treatment couch. This process of image-guidance has been shown to improve the accuracy of radiotherapy treatment at multiple treatment sites [7-9]: the major advantage of the CBCT system is kilovoltage (kV) volume imaging with sufficient soft-tissue contrast to visualize the target itself [10]. This allows detection and correction of internal target position errors, which are independent of the bony anatomy.

However, not only spatial changes of the target position are seen in these verification images. In conventionally fractionated radiotherapy, regression of the treated macroscopic tumours has been observed especially for head and neck tumours and lung cancer [11,12]. Adaption of radiotherapy treatment to such changes of the target volume is currently being discussed intensely. The use of one-beam CT images for adaptive radiotherapy would avoid repeated spiral CT imaging in addition to images acquired for image-guidance. Avoiding excessive radiation dose to the patient for image acquisition and reduced work-load are consequences if CBCT images can be used for treatment planning and dose calculation. Letourneau et al. presented an approach to use cone-beam CT images for target definition, online planning and efficient process integration [13]. For accurate dose calculation based on CBCT images, the relationship between Hounsfield units (HU) and density (D) is required. Several authors have investigated the suitability of the CBCT for dose calculation and developed different pixel correction strategies depending on the CBCT system properties [11,13-23]. Megavoltage (MV) and kV CBCTs offer different image performance with regard to soft tissue contrast, scatter radiation and image acquisition settings [11,21].

The CBCT imaging technique and its acquisition parameters influence the image quality by the amount of radiation scattered at the level of the flat panel. Image acquisition for CBCT can be modified by tube voltage, collimation, filter type, half and full fan mode. A change of the acquisition parameters for kV CBCT affects the image quality and pixel value distribution. In addition, the magnitude of scatter and artefacts are affected by the scanned object size [22-24], CT value fluctuation due to a change in scatter irradiation [22]. Compared to planning CTs, a reduced number of projections is acquired and less information is available for image reconstruction [17]. The CT values of a CBCT cannot directly be used for dose calculations, because this might lead to inaccurate dose calculations [21].

Basic procedures for relating CT values to radiological parameters and implementing them in treatment planning systems have been described by several authors [25,26]. Several methods are described in the literature to improve image quality of CBCT images [11,13,18,20,21]. Pixel correction strategies range from look-up-tables, histogram matching [15,18,20] to pixel calibration based on phantom measurements [11,20,22,26,27]. Zijtveld et al. described a method to map HU from planning CT to CBCT based on a rigid registration algorithm [21] to account for deformation of the planning and CBCT. A reference CT is needed to compare and correct the CT values for all methods using registration procedures. Currently, two systems offer CBCT for image-guided radiotherapy: Elekta XVI and Varian OBI. There are differences between these two systems regarding their usability for dose calculation. To date most investigations were based on the Varian OBI system. The investigation of Varian OBI system showed only small differences in density calibration between planning and CBCT (less than 10 HU) which makes it easily available for treatment planning [17,23]. In contrast, the Elekta Synergy CBCT system XVI showed larger deviations in HU which makes correction strategy necessary [16,21].

This work analyses the suitability of the Elekta CBCT system for dose calculation. The impact of CBCT number accuracy and reproducibility on dose calculation performed with these images is investigated for phantom data and three patient populations.

## Methods

Two CT volume imaging systems were used for image acquisition and dose calculation: the planning CT (Somatom Sensation Open, Siemens, Forchheim, Germany) as a reference and the CBCT (Synergy XVI, Elekta, Crawley, UK). Both systems operate with tube voltages ranging from 80 to 140 kV. Images with the conventional helical CT scanner were acquired with the standard presets of the manufacturer.

For CBCT imaging, the kV panel can be positioned laterally (using motorized movements) at three different 'field of view' (FOV) positions: S, M and L. In the medium FOV position (M), the centre of the kV detector panel is offset by 115 mm from the kV central axis and then a full fan rotation (360°) is necessary for complete image acquisition. If the panel position is placed in small FOV position (S), the kV central axis is equal to the panel centre and a half fan is sufficient [28]. The bow tie filter (F1) is a kV filter which is inserted between the X-ray source and the patient to reduce intensity variations across the detector [23]. For S20F0 the cone beam data set was acquired with flat panel parked in position S, no filter (F0) was inserted and 20 cm longitudinal extension.

First the impact of scan parameters (tube voltage, filtering and collimation) on CT values in the CBCT images was evaluated for phantom measurements, pixel correction strategies were developed and dose calculation was performed on a rigid phantom geometry. Afterwards dose calculations were performed in CBCT studies of real patients. The patient data was initially required for verification of the treatment position and was retrospectively evaluated for dose calculation.

### Phantom measurements

**Figure 1:**
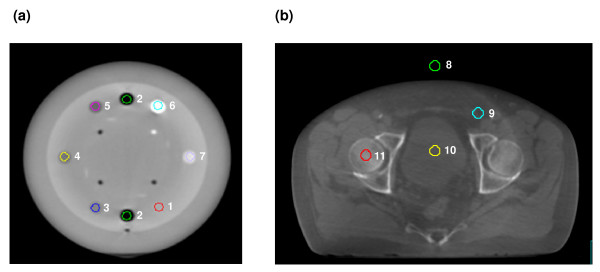
(a) Axial slice of CATPhan^® ^geometry acquired with the CBCT system and volumes of interest for each material insert: 1 Acrylic, 2 Air, 3 Polystyrene, 4 LDPE, PMP, 6 Teflon^®^, 7 Delrin^®^. (b) Example of volume definition for a pelvis patient: 8 air, 9 fat, 10 fluid, 11 femoral head.

Phantom measurements were performed to eliminate CT value variations due to deformation processes during radiotherapy treatment. In the first part, CT and CBCT images of 2 phantom geometries were acquired and compared with respect to their suitability for pixel value calibration: CATPhan^® ^(CATPhan^® ^CTP503, Phantom Laboratory, Salem, NY) and Gammex RMI^® ^(Gammex RMI 467^®^, Gammex RMI, Middleton, WI). The Gammex RMI^® ^was used to establish the relationship between the density of different materials and their corresponding CT values for the planning CT. The body of the CATPhan^® ^contains seven different material inserts listed in Table 1. The influence of various scan parameters (tube voltage, filter and collimator, rotation angle) on the CT values was investigated using 6 different CBCT presets (M20F1 120 kV, M10F1 120 kV; S20F0 120 KV 40mA, S20F0 120 KV 25 mA, S10F0 100 kV, S20F0 100 kV). Volumes of interest (VOI) were defined (figure 1a) in regions of uniform density in the CATPhan^® ^and the corresponding mean CT values were measured in the planning CT and the CBCT data sets. Based on these measurements, the density calibration tables (HU-D table) were determined. The ADAC Pinnacle treatment planning system (TPS) v7.6s (Philips/ADAC, Milpitas, CA, USA) was used for contouring and dose calculation, details about definition of HU-D table were previously described by Saw et. al [26].

**Table 1:** Phantom inserts.

**Material**		**specified density (g/cm^3^)**	**HU-D_pCT _density (g/cm^3^)**	**HU-D_pCT _CT value (HU)**
Air		0	0	0
PMP	C_6_H_12_(CH_2_)	0.83	0.81	800
LDPE	C_2_H_4_	0.92	0.91	900
Polystyrene	C_8_H_8_	1.05	0.96	960
Acrylic	C_5_H_8_O_2_	1.18	1.12	1120
Delrin^®^	proprietary	1.41	1.27	1340
Teflon^®^	CF_2_	2.16	1.61	1950

CATPhan^® ^material inserts and their specified density values are listed; for comparison the measured CT values in the planning CT and the corresponding density determined from the Gammex RMI^® ^table HU-D_pCT_.

### Phantom pixel correction

Based on the phantom measurements with different CBCT imaging parameters, different pixel correction strategies were developed which are listed below (see also table 2):

**Table 2:** Phantom measurements.

	**1F**		**4F**		**IMRT plan**
**Correction strategy**	**6 MV**	**18 MV**	**6 MV**	**18 MV**	**10 MV**

**(a) CBCT, S10F0**					
					
HU-D_pCT_	19.9% ± 2.5%	15.5% ± 2.1%	19.5% ± 0.7%	14.7% ± 0.5%	8.8% ± 6.3%
HU-D_S10F0_	4.2% ± 1.2%	2.6% ± 0.9%	1.8% ± 0.3%	2.1% ± 0.3%	0.8% ± 0.8%

**(b) CBCT, M20F1**					
					
HU-D_pCT_	18.4% ± 3.7%	13.7% ± 2.4%	12.7% ± 1.0%	11.6% ± 0.5%	6.8% ± 5.6%
HU-D_M20F1_	2.6% ± 0.7%	1.9% ± 0.9%	3.2% ± 0.7%	1.1% ± 0.3%	1.0% ± 1.1%

Comparison of dose calculation based on planning CT and CBCT with different correction strategies for the different test plans (1F, 4F and IMRT) for phantom studies acquired with different presets: (a) collimation S10 and no filter (S10F0) and (b) collimation M20 and bow tie filter (M20F1). The standard table (HU-D_pCT_) was created based on the CT and densities values of the planning CT phantom scan. The phantom based tables HU-D_M20F1_, HU-D_S10F0 _were based on the CBCT phantom data sets acquired M20F1 and S10F0, respectively. The mean difference between the dose in the planning CT and the CBCT is given in percentage of the dose based on the planning CT.

- A standard HU-D table (HU-D_pCT_) was established for the Siemens Somatom CT scanner for both phantoms (Gammex RMI^® ^and CATPhan^®^). The mean CT values were determined in the planning CT for each VOI, i.e. for each material insert and the corresponding density values were determined based on the relationship between CT values and physical density as specified by the phantom manufactures.

- HU-D tables were generated from the phantom CBCT datasets of the CATPhan^® ^separately for the different image acquisition parameters: the mean CT values within each material insert (e.g. CBCT#_Air_, CBCT#_PMP_) were measured and the corresponding density values of the planning CT described the CBCT phantom HU-D table. HU-D_M20F1 _is specific for CBCT images acquired with collimation M20, bow tie filter F1 and tube voltage 120 kV. The procedure was repeated for the preset S10F0 which resulted in a second HU-D table (HU-D_S10F0_).

### Phantom dose calculation

The dose distributions in planning CT and CBCT of the CATPhan^® ^were compared by applying three different isocentric plans (one field (1F), four fields (4F) and clinical seven field IMRT plan) to the planning CT first. The beam arrangements including the calculated monitor units were then transferred to the CBCT phantom geometry. For 1F und 4F techniques, the mean doses within the contoured VOIs (figure 1a) were compared between dose calculation in the planning CT and CBCT. Dose deviation was expressed by the mean of absolute differences and its standard deviation within the VOIs. For the IMRT beam arrangement dose distributions were compared with orthogonal dose planes. For dose plane comparison dose difference was evaluated in a region of interest surrounding the high dose region.

### Measurements in real patients data-sets

The second part of this study is based on data sets of 33 patients (11 prostate cancer, 11 head tumour and 11 thorax patients): variability of CT values and the accuracy of dose calculation was investigated in these CBCT data sets. According to our clinical protocol, CBCT images of thorax and pelvis patients were acquired with the following scanning parameters: collimation M20, bow tie filter F1 120 kV and rotation angle 360°. A further CBCT preset was used for image acquisition of the head patients: collimation S10, no filter (F0) and rotation angle of 180°. Corresponding planning CT datasets were taken for comparison.

Depending on the tumour location and scan volume, different VOIs in areas of nearly homogenous density were defined in the planning CT and CBCT. For pelvis patients air, fat, fluid, symphysis, femoral head and femur were delineated; the position of these VOIs was in fixed relationship to the bony anatomy (Figure 1b). In the thorax scans the VOIs were contoured in air, lung, fat, blood, muscle, bone and cortical bone. In the data sets of the head patients, contours were defined for air, neck support, eye, brain and skull. The mean CT values of these VOIs in the planning CT were used as reference for generation of the HU-D tables.

In addition, organs were contoured for dose comparison from a clinical point of view. The clinically treated radiotherapy plans were recalculated employing the CBCT data sets and doses to target volumes and organs-at-risk were compared by means of dose-volume histogram (DVH) comparisons. If the beam arrangement for head patients included non-coplanar beams and the CBCT patient model was not complete at the superior end, we excluded these patients from the patient plan evaluation. Some of the thorax and pelvis patients showed incomplete patient models because the body contour exceeded the FOV of the CBCT- these patients were excluded as well. The following volumes were contoured in the data sets of the thorax patients: CTV, PTV, oesophagus, spinal cord, heart and ipsilateral lung. The contours of PTV, PTV Boost, PTV Ring1, PTV Ring2, bladder and rectum were delineated for the pelvis patients. For head patients PTV, PTV Ring1, PTV Ring2, chiasm and brainstem were contoured. Two rings (PTV Ring1, PTV Ring2, each having a radial extend of 1 cm) were generated around the PTV to consider the dose gradient for dose comparison. The mean dose, D05 and D95 were compared for target volumes, D01 for organs at risk and the mean dose for the ipsilateral lung.

### Patient pixel correction

Different HU-D tables were used for the dose calculation in the clinical CBCT data sets (see table 3):

**Table 3:** Patient measurements.

		**1F**		**4F**		**patient plan**	
**Correction strategy**		**6 MV**	**18 MV**	**6 MV**	**18 MV**	**dose plane**	**DVH**

**(a) Pelvis Patients**							
						
standard	HU-D_pCT_	21.6% ± 3.7%	15.7% ± 3.4%	14.1% ± 2.1%	10.5% ± 1.7%	8.0% ± 5.7%	19.1% ± 3.4%
phantom based	HU-D_M20F1_	7.7% ± 5.2%	5.6% ± 3.9%	11.2% ± 3.7%	8.4% ± 2.9%	5.2% ± 3.7%	12.7% ± 1.5%
group based	HU-D_Pelvis_	2.7% ± 2.3%	2.4% ± 2.0%	2.2% ± 1.9%	1.9% ± 1.5%	0.9% ± 0.9%	1.3% ± 1.0%
patient based	HU-D_Pat_i_	2.4% ± 1.7%	2.0% ± 1.4%	1.6% ± 1.3%	1.2% ± 1.1%	0.9% ± 0.9%	1.2% ± 0.9%

**(b) Thorax Patients**							
						
standard	HU-D_pCT_	21.6% ± 9.6%	16.4% ± 7.2%	17.1% ± 5.8%	12.6% ± 4.6%	10.9% ± 6.8%	13.8% ± 10.5%
phantom based	HU-D_M20F1_	11.5% ± 7.1%	8.9% ± 5.4%	10.4% ± 4.6%	7.7% ± 3.7%	8.1% ± 3.7%	6.2% ± 4.7%
group based	HU-D_Thorax_	4.1% ± 3.5%	3.2% ± 2.8%	3.2% ± 2.2%	2.6% ± 1.9%	1.7% ± 1.7%	1.7% ± 2.4%
patient based	HU-D_Pat_i_	4.0% ± 3.4%	3.0% ± 2.7%	2.9% ± 1.9%	2.3% ± 1.7%	1.8% ± 1.6%	1.7% ± 2.0%

**(c) Head Patients**							
						
standard	HU-D_pCT_	22.4% ± 10.2%	14.9% ± 9.7%	19.1% ± 4.6%	10.9% ± 5.8%	14.5% ± 10.4%	16.2% ± 12.7%
phantom based	HU-D_S10F0_	3.0% ± 2.7%	2.5% ± 1.0%	2.3% ± 1.9%	2.0% ± 1.7%	1.4% ± 2.4%	1.6% ± 2.2%
group based	HU-D_Head_	2.1% ± 1.7%	2.1% ± 1.5%	1.6% ± 1.4%	1.5% ± 1.5%	1.3% ± 2.3%	1.4% ± 1.9%
patient based	HU-D_Pat_i_	2.1% ± 1.7%	1.9% ± 1.5%	1.5% ± 1.3%	1.4% ± 1.4%	1.5% ± 2.5%	1.4% ± 1.9%

Comparison of dose calculation based on planning CT and CBCT with different correction strategies for the different test plans (1F, 4F and IMRT) for (a) pelvis, (b) thorax and (c) head patients. The mean difference between the dose in the planning CT and the CBCT is given in percentage of the dose based on the planning CT. The standard table (HU-D_pCT_) was generated from CT and density values measured in the planning CT. The phantom based tables HU-D_M20F1_, HU-D_S10F0 _were based on the CBCT phantom data sets acquired with collimation M20/bow tie filter and collimation S10/no filter, respectively. The group based tables represent the mean HU-D tables (HU-D_Pelvis_, HU-D_Thorax_, HU-D_Head_) for the specific patient population (pelvis, thorax, head) while the individual tables (HU-D_Pat_i_) were created based on each patient data set. Dose differences were calculated in volumes of interest for 1F and 4F techniques. For patient plan the dose was compared based on dose planes and dose volume histograms (DVH).

- The standard table HU-D_pCT _was based on planning CT images previously described for the phantom study. No adjustment of this table for CBCT imaging was performed. Density values for patient specific VOIs (air, fat, fluid ...) in the CATPhan^® ^were taken from the relation ship in HU-D_pCT _in the Gammex RMI^® ^density phantom.

- Acquisition parameter specific tables HU-D_M20F1 _and HU-D_S10F0 _were based on CBCT images of the CATPhan^® ^as described above. HU-D_M20F1 _was used for dose calculation in the CBCT data sets of the thorax and pelvis patient. HU-D_S10F0 _was applied to the CBCT data sets of the head patients.

- Tables were generated separately for the three different patient groups head, thorax and pelvis (HU-D_Pelvis_, HU-D_Thorax _and HU-D_Head_): these tables were based on the mean CBCT value of each patient population. The CT values were taken from the patient CBCT data sets and density for each VOI was determined in the planning CT and was listed to the corresponding CBCT value.

- Patient individual tables (HU-D_Pat_i_) were created separately for each patient: the mean CBCT value for each VOI in the patient CBCT data set was calculated and this was allocated to the corresponding density of the planning CT.

### Patient dose calculation

According to the phantom study, the same beam arrangements were applied to both image sets (planning CT and CBCT) and differences in the dose distribution were analysed. For incomplete patient models in the thorax population, a two beam technique was used instead of 4F. Comparisons with real patient plans were performed for 5 pelvis, 5 thorax patients and 5 head patients. Treatment plans of the head patients consisted of three-dimensional conformal plans with 4 to 9 beams and 6 MV photon energy. Treatment plans of the thorax patients consisted of three-dimensional conformal plans with 6 to 9 beams of 6 and 10 MV photon energy. All plans of the pelvis patients were based on IMRT for prostate cancer with 7 beams; photon energy was 10 MV, the number of step-and-shoot segments ranged from 30 to 50.

Planning CT and CBCT image sets were registered and then the plan geometry was transferred to the CBCT without any changes, i.e. the number of total monitor units remained unchanged. The dose was recalculated in the CBCT based on the four different pixel correction strategies described above. The dose distributions were evaluated by DVH and dose planes. Orthogonal dose planes were calculated in the coronal and sagittal orientation with a source-plane-distance of 100 cm. For the IMRT techniques, the dose planes were analysed using Scanditronix, Omni Pro-IMRT RT1.5 software (Scanditronix-Wellhöfer/IBA, Uppsala, Sweden). Dose differences between dose planes were quantified by the absolute mean difference within a region of interest which was limited to the high dose area. Because of organ deformation between image acquisition of the planning CT and the CBCT, additional contours were delineated in the CBCT data set and included in DVH evaluation: for pelvis patients, rectum or bladder were recontoured and for thorax patients the lung was delineated in the CBCT image sets.

## Results

### Phantom Study

**Figure 2:**
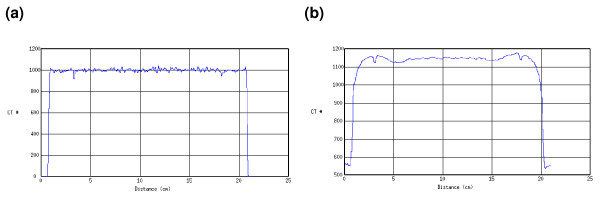
Intensity profile in an axial CT slice for the CATPhan^® ^geometry acquired with the planning CT (a) and CBCT system (M20F1) (b).

The image quality and the CT values of the CBCT data set were different to the planning CT. This is illustrated in figure 1. Steep gradients in CT values, for example the peripheral contour of the phantom, were less steep in the CBCT data set than in the planning CT (figure 2). For the profile of the CBCT, less high frequency variation of the CT values was observed and the phantom edge appeared low pass filtered compared to the planning CT. Due to the reduced number of projections (400 – 700) for image reconstruction the CBCT offered a limited image quality compared to the planning CT (2000 – 4000 projections).

**Figure 3:**
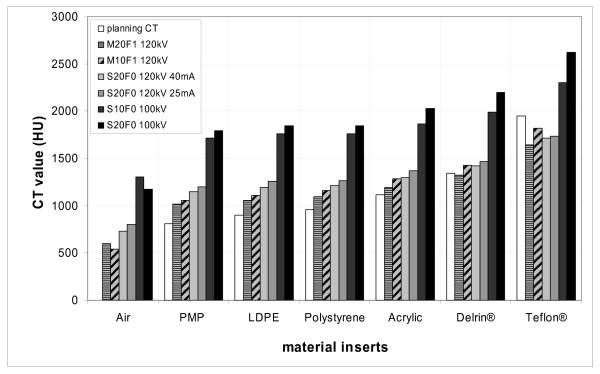
Variation of CT values in Hounsfield units (HU) for seven different materials measured in the planning CT and the CBCT. Six presets for CBCT image acquisition were compared. Large variations in HU were observed for each material insert depending on the CBCT acquisition parameters.

**Figure 4:**
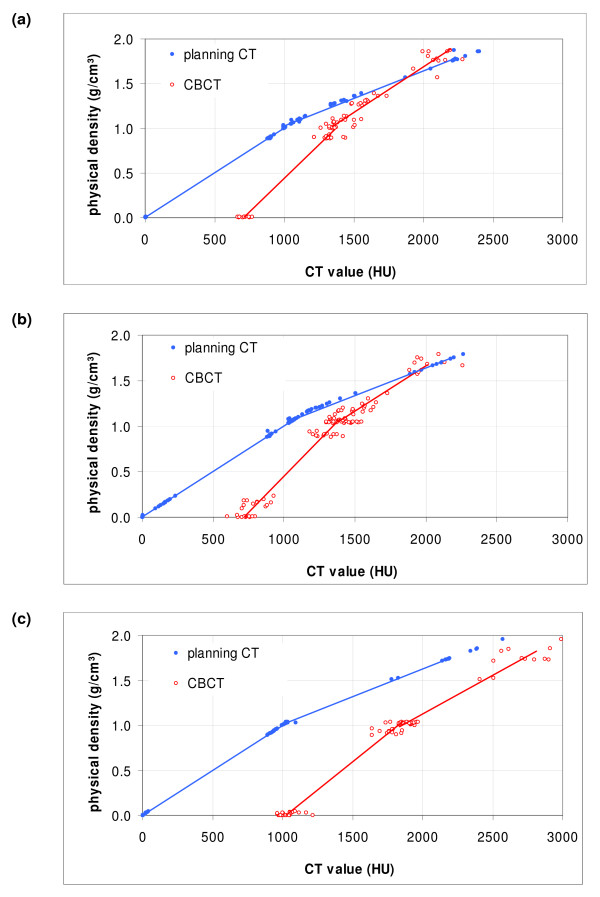
Generated HU-D tables for planning CT (blue filled circles) and the group based CBCT (red open circles) based on three patient populations: (a) pelvis patients, (b) thorax patients and (c) head patients.

The CT values in the CBCT were determined for the most frequently used CBCT acquisition parameters. A wide variation of CT values within the isodense VOIs was observed depending on image acquisition parameters of the CBCT tube voltage, collimation and filter type. The variation was evident for all seven subvolumes (material inserts) in the phantom (564 HU ± 377 HU). Figure 3 shows the mean CT value for each material insert depending on the six different presets for CBCT acquisition. For all presets, the largest difference between planning CT and CBCT was observed for the air-insert. The CT value in the planning CT was nearly zero while the pixels in the CBCT were ranging from 540 to 1300 HU depending on the preset type. For the presets M20F1 and S10F0, the difference in CT values was reduced for denser materials like Delrin^® ^and Teflon^® ^(Figure 4).

The effect of different pixel correction strategies for dose calculation was investigated. The HU-D tables were interpolated bilinearly. For the standard table HU-D_pCT_, the CT values were determined in CATPhan^® ^and Gammex RMI^® ^geometry. Both resulting tables for HU-D_pCT _were in good agreement for densities ranging from 0 – 1.18 g/cm^3 ^(Air, polymethylpentene PMP, low-density polyethylene LDPE, Polystyrene and Acrylic). The measured CT values for Delrin^® ^and Teflon^® ^(1340 HU and 1990 HU) were in agreement with the literature values but the specified density values (1.41 g/cm^3 ^and 2.16 g/cm^3^) did not agree with the HU-D table of Gammex RMI^®^. Gammex RMI^® ^is especially made for electron density calibration and contains tissue equivalent materials (Brain, Bone, Liver) while the CATPhan^® ^is made of tissue substitutes (Acryil, Delrin^®^, Teflon^®^). Schneider et al obtained different HU-D tables depending on calibration material: Mylar/Melinex/Teflon^® ^and biological tissue inhomogeneities [29]. For the current investigation, we established the HU-D_pCT _relationship based on the Gammex RMI^®^. Dose calculations in the CBCT of the CATPhan^® ^were performed with HU-D_pCT_, HU-D_M20F1 _and HU-D_S10F0 _and dose distributions were compared with the planning CT. The results for the phantom geometry are listed in table 2. For all simple field arrangements (1F and 4F), the accuracy of dose calculation was not acceptable for HU-D_pCT_. Results were considerably improved for the preset based correction strategy (HU-D_M20F1 _and HU-D_S10F0_). If 1F 6 MV was applied to the phantom images acquired with S10F0 and M20F1 using the corresponding preset based tables (HU-D_S10F0_, HU-D_M20F1_) the mean difference in dose was reduced from 19.9% ± 2.5% and 18.4% ± 3.7% to 4.2% ± 1.2% and 2.6 ± 0.7%. Larger deviations were found for the 1F techniques than for the 4F techniques because of the location of the VOIs within areas of the inhomogeneous dose distribution for the 1F technique. For the IMRT technique, the dose deviation was determined by evaluating the dose planes within a region of interest surrounding the high dose region. IMRT dose calculation in the phantom geometry (displayed in figure 1) showed the largest deviation for HU-D_pCT_: 8.8% ± 6.3% and 6.8% ± 5.6% for CBCT images acquired with presets S10F0 and M20F1. When the preset based tables (HU-D_S10F0_, HU-D_M20F1_) were applied for dose calculation the deviations decreased and the accuracy of dose calculation was improved to differences of 0.8% ± 0.8% and 1.0% ± 1.1%.

### Patient study

The HU-D tables calculated for the three patient groups (pelvis, thorax and head) and the standard HU-D_pCT _are shown in figure 4. For the planning CT, the mean CT values within the VOIs and the corresponding density values are plotted with filled circles: identical HU-D_pCT _relationship was observed for the pelvis, thorax and head patient group. The density values of the planning CT were taken as reference for calibration of the CBCT curves. The mean regression curve of the CBCT data defined the patient group based tables HU-D_Pelvis_, HU-D_Thorax _and HU-D_Head_. The planning CT data shows CT values ranging from 0 to 2400 HU while the CBCT offers a limited data range from 666 to 2484 HU. The HU-D table was interpolated bilinearly. This was based on the finding of several investigators [26,27]. The quality of the bilinear fit was estimated by the coefficient of determination (COD). The data fits for the generated tables (HU-D_pCT_, HU-D_Pelvis_, HU-D_Thorax _and HU-D_Head_) showed good correlation with COD between 0.99 and 1.

Table 3 summarizes the comparison of the dose distributions in the planning CT and the corresponding CBCT for three patient populations using the four different pixel correction strategies described above. The largest dose deviation was observed with 1F 6 MV using the standard CT table HU-D_pCT_: differences to dose distributions in the planning CT were 21.6% ± 3.7%, 22.4% ± 10.2% and 21.6% ± 9.6% for the pelvis, thorax and head patients. Application of CATPhan^® ^tables HU-D_M20F1 _and HU-D_S10F0 _improved dose calculation accuracy in the CBCTs significantly: differences were about 10% for the pelvis and thorax group compared to differences less than 5% for the head group. The use of patient and group based HU-D tables resulted in small dose differences between planning CT and CBCT: differences were less than 5%. Nearly the same precision in dose calculation was achieved with the averaged group tables (HU-D_Pelvis_, HU-D_Thorax _and HU-D_Head_) compared to patient specific tables HU-D_Pat_i_.

**Figure 5:**
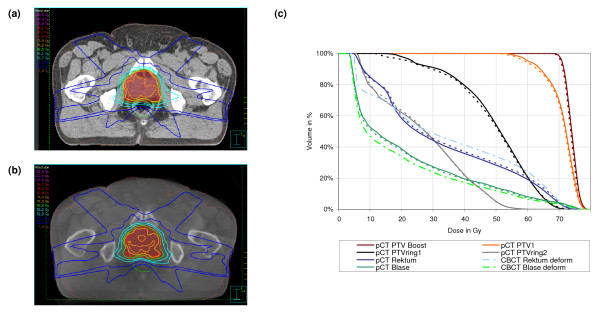
Isodose distribution in axial slices for IMRT technique of a pelvis patient: (a) dose calculation based on planning CT and standard HU-D table and (b) dose in CBCT using the patient based HU-D table and (c) DVH for the contoured ROIs in the planning CT (solid) and CBCT (dashed). An additional rectum volume was contoured and evaluated in the DVH (dashed-dotted) due to a reduced rectum filling in the CBCT data set.

**Figure 6:**
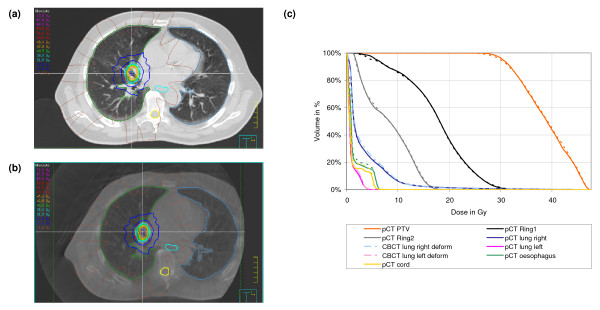
Isodose distribution in axial slices for IMRT technique of a thorax patient: (a) dose calculation based on planning CT and standard HU-D table and (b) dose in CBCT using the patient based HU-D table and (c) DVH for contoured ROIs in the planning CT (solid) and CBCT (dashed). The lungs were recontoured in the CBCT data set and included in the DVH evaluation (dashed-dotted).

**Figure 7:**
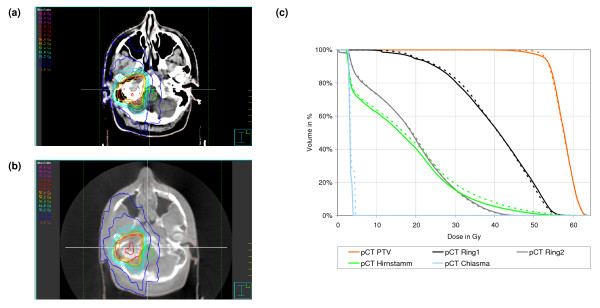
Isodose distribution in axial slices for IMRT technique of a head patient: (a) dose calculation based on planning CT and standard HU-D table and (b) dose in CBCT using the patient based HU-D table and (c) DVH for contoured ROIs in the planning CT (solid) and CBCT (dashed).

Dose distribution and DVH for planning and CBCT are shown in figures 5, 6 and 7 for one selected patient of each population. These dose calculations were based on the patient specific HU-D tables (HU-D_Pat_i_). DVH comparison showed good correlation between the calculated doses in planning and CBCT: mean dose differences was 1.20% ± 0.91%, 1.72% ± 0.99% and 1.36% ± 1.96% in all contoured volumes for pelvis, thorax and head patients. The dose distribution in the ring contours around the PTV was similar for planning CT and CBCT which implies the same dose gradient around the PTV. The dose plane evaluation showed small deviations: 0.9% ± 0.9%, 1.8% ± 1.6% and 1.5% ± 2.5% for pelvis, thorax and head patients.

Figure 5 shows the comparison of the IMRT dose distribution for one pelvis patient using the patient specific table HU-D_Pat_i_: differences in the dose planes were small with 0.7% ± 0.5% for the selected patient. Almost the same accuracy was attained by using the patient group based table HU-D_Pelvis_. Changes in rectum and bladder filling at the time of CBCT image acquisition for the selected patient necessitated an additional rectum and bladder contour delineation in the CBCT and evaluation in the DVH. An increased volume of the adjusted rectum in the CBCT was exposed to doses larger than 20 Gy and simultaneously the low dose region was reduced. The bladder volume was increased by 30 cm^3 ^which resulted in a mean dose reduction of 11%.

Dose comparison for one thorax patient is shown in figure 6 based on the patient specific table HU-D_Pat_i_. Despite an incomplete patient model, the patient was selected for dose comparison because no beam entered the patient at this site. The patient was treated with an extracranial stereotactic technique which was transferred to the CBCT. Only small differences were detected in the DVH for the deformed lung volumes. The changes in lung volume were 356 cm^3 ^and 340 cm^3 ^for the left and right lung, respectively which increased the mean dose by 3% in the CBCT.

Figure 7 shows the dose distribution in planning and CBCT for a head patient. We assumed there was no organ deformation for the head patient population. The dose was based on a 5 field IMRT technique with 30 step-and-shoot segments. The use of phantom, group or patient based HU-D tables resulted in similar precision of 1.3% to 1.6% for the 5 selected patients of the head population. The surface of the head was more precise in the planning CT than in the CBCT. Consequently, we observed larger dose deviations near patient outline due to the smoother pixel gradient in the CBCT.

## Discussion

Recent progress in imaging and radiotherapy treatment planning has made adaptive radiotherapy a focus of research. Its aim is to adjust the radiotherapy treatment plan to changes occurring during the course of treatment: regression of the tumour due to radio (chemo-) therapy and loss of patient weight are considered to be the most significant. Adaptive radiotherapy requires frequent and repetitive imaging of the patient to visualize and quantify these changes. Using CBCT studies, which were acquired for image-guidance, for plan adaptation is a logical step to keep patient radiation dose and work-load within acceptable limits. Consequently, it was the aim of this study to establish techniques for accurate dose calculation in CBCT studies.

Large deviations of CT values between planning CT and CBCT were observed: this was similar for phantom and clinical CBCT studies. This is in agreement with data from Zijtveld et al. and Yang et al. [21,22]. In consequence the use of HU-D_pCT _is associated with unacceptable inaccurate dose calculation in the CBCT studies. Additionally, CT values were highly influenced by the CBCT image acquisition parameters tube voltage, filtering and collimation. This suggests that a single HU-D table will not be applicable to different imaging presets, as used for head or pelvis CBCT imaging for example. As a consequence we developed specific HU-D tables for the two CBCT image acquisition presets, which are most frequently used in our clinical practice.

The use of phantom based HU-D tables (HU-D_M20F1_, HU-D_S10F0_) resulted in small errors for CBCT dose calculation in the cranial region. This is explained by a similar geometry and size of the CATPhan^® ^compared to the patients heads. However, these phantom based HU-D tables were inaccurate for thorax and pelvis patients resulting in errors larger than 5%. This clearly shows the influence of the patient geometry on CT values in the CBCT and the subsequent influence on dose calculation. The influence of the scan object size was previously investigated by Yang et al who observed an increasing scatter contribution for larger objects [22]. This is borne out by our results: we observed higher CT values for outside patient air with increasing body size.

We observed only small differences between CBCT dose calculation with HU-D tables specific for the patient group or specific for each individual patient: errors were less than 5%. Consequently, the generation of three different HU-D tables would be sufficient for accurate dose calculation in the head, thorax and pelvis region. Furthermore, we expect applicability of HU-D_Head _for CBCT dose calculation in the head and neck region and HU-D_Pelvis _for dose calculation in the abdominal region.

Once the patient group based HU-D tables are created, no reference data set (such as a planning CT) is needed for comparison and/or rescaling of the pixel value. For the presented approach, no segmentation of the CBCT [14,19] for rescaling of the pixel values is necessary. The dose calculation can be performed directly on the CBCT dataset and no additional software tool is needed for dose calculation. The method requires the possibility in the TPS to create individual HU-D tables for dose calculation based on CBCTs, which is not the case for all TPS (some TPS use a formula to calculate the density from the CT numbers instead of lookup tables).

We could not confirm the findings of Yoo et al. and Lee et al. who observed only small differences between HU values of CBCT and planning CT based on the OBI system [17,23]. Parker stated, that 5% uncertainty in determination of CT scanner density will result in deviation of 1% in the calculated dose [27]. The comparison of HU in planning CT and CBCT (M20F1) showed average differences of 40% for density 1 g/cm^3^. Uncertainty of 40% in determination of CT scanner density resulted in dose plane deviation of 8% for IMRT cases of the pelvis population without any correction of CT values. This is in agreement to the findings of Parker et al..

Users of the Varian OBI system who developed correction strategies reported dose deviation of 3% [17] for phantoms and 5% [17], 1% [22], 0.3% [15] for patient data. Pixel correction of images acquired with the Elekta XVI system resulted in dose differences of less than 1% [16,21]. We achieved an accuracy of nearly 1 – 2% on average which is comparable to the published data.

It should be noted that a full acquisition of the patient model is needed to allow a complete dose calculation in the process of adaptive radiotherapy. If some parts of the patient model are out of the FOV in the CBCT, a reference image set with the whole information is necessary to replace missing pixels as demonstrated by van Zijtveld et al. [21]. In our study we typically observed incomplete patient models for thorax patients. Most of these patients were treated with stereotactic body-radiotherapy for peripheral lesions. Consequently, the treatment isocentre was not in the centre of the patient but in the centroid of the peripheral lesion. Incomplete patient models were acquired due to the FOV size of 41 cm in diameter for the M collimation. The L collimation offers a larger FOV and would ensure a complete acquisition of the patient model; however, image quality is significantly decreased with this large FOV. Currently, the M collimation is used in our clinical protocol for thorax patients as a compromise between image quality and FOV.

We found that the slope of the HU-D table varied depending on the tube voltage. The CBCT data sets were additionally influenced by collimation and filter type. Therefore it is important to ensure that for the generation of the group based tables HU-D (HU-D_Pelvis_, HU-D_Thorax_, HU-D_Head_) all data of the used patient population is acquired with the same parameters and is consistent with parameters used for the examined dataset for dose calculation.

The presented pixel correction strategies were based on look-up-tables and did not consider any organ deformation. The time span between the acquisition of planning CT and CBCT was about 3–5 days and organ deformation can occur due to variations in organ fillings. Yang showed that non-rigid image registration is a useful tool to consider organ deformation between the reference and the CBCT data set [22]. Additionally, such deformable image registration will be necessary in the further process of adaptive radiotherapy.

## Conclusion

A correction of CT values was necessary for dose calculation with the cone beam data sets. The best accuracy of dose calculation in CBCT images was achieved with patient specific HU-D table; however differences to patient group specific HU-D tables were clinically negligible. Three HU-D tables specific to CBCT image acquisition parameters and specific to anatomical regions pelvis, thorax and head and neck are considered to be sufficient. Once the group based HU-D tables are created, direct dose calculation on CBCT datasets is possible without the need of a reference CT for pixel value calibration.

## Competing interests

Presented in part at the Congress of "Deutschen Gesellschaft für Radioonkologie" (DEGRO – ÖGRO) 2008, Vienna, Austria.

## Authors' contributions

All authors read and approved the final manuscript. AR designed the study and the analysis, performed the simulations and revised the manuscript. QH participated in data collection and analysis. DS participated in data collection and analysis. KB participated in the study design and revised the manuscript. JW revised the manuscript. MG revised the manuscript. MF revised the manuscript.
